# Retraction: RNAi-mediated silencing of vitellogenin gene curtails oogenesis in the almond moth *Cadra cautella*

**DOI:** 10.1371/journal.pone.0275947

**Published:** 2022-10-21

**Authors:** The PLOS ONE Editors

The *PLOS ONE* Editors retract this article [1] because it was identified as one of a series of submissions for which we have concerns about authorship, competing interests, and peer review. We regret that the issues were not addressed prior to the article’s publication.

All authors did not agree with retraction.

## References

[1] Husain M, Rasool KG, Tufail M, Alwaneen WS, Aldawood AS (2021) RNAi-mediated silencing of vitellogenin gene curtails oogenesis in the almond moth *Cadra cautella*. PLoS ONE 16(2): e0245928. 10.1371/journal.pone.0245928 33571307 PMC7877660

